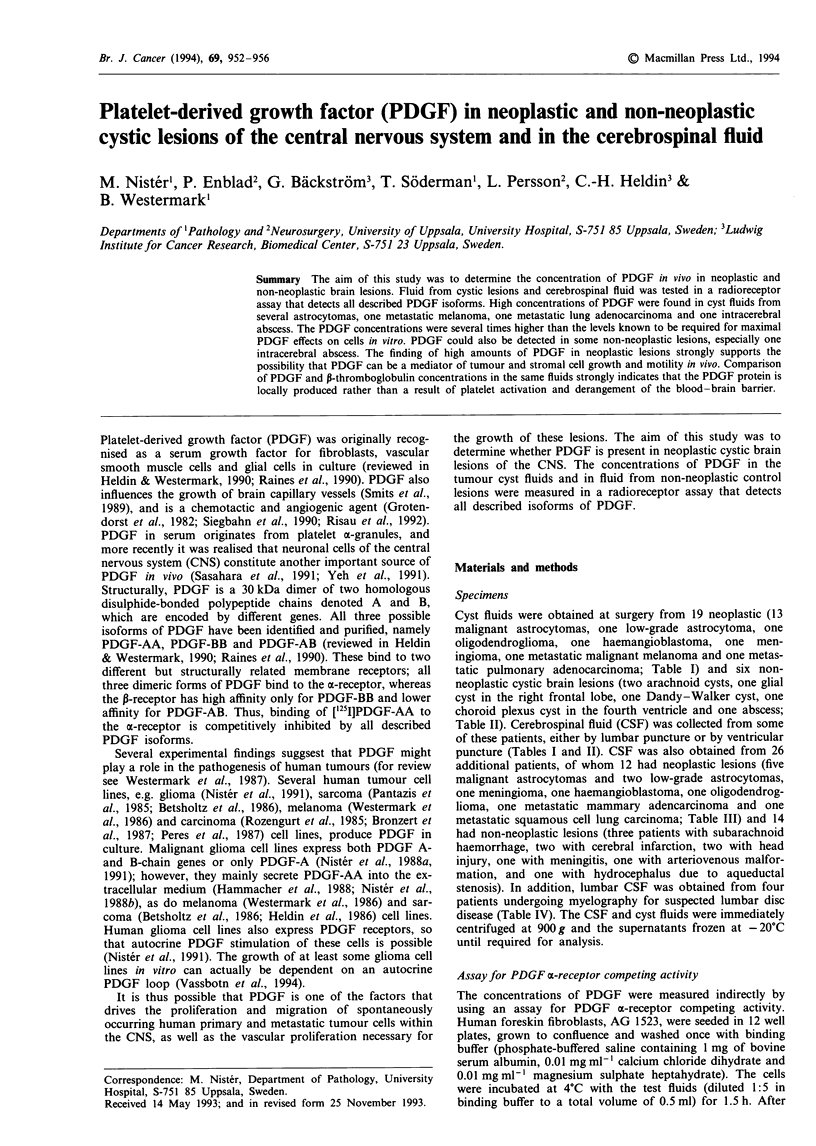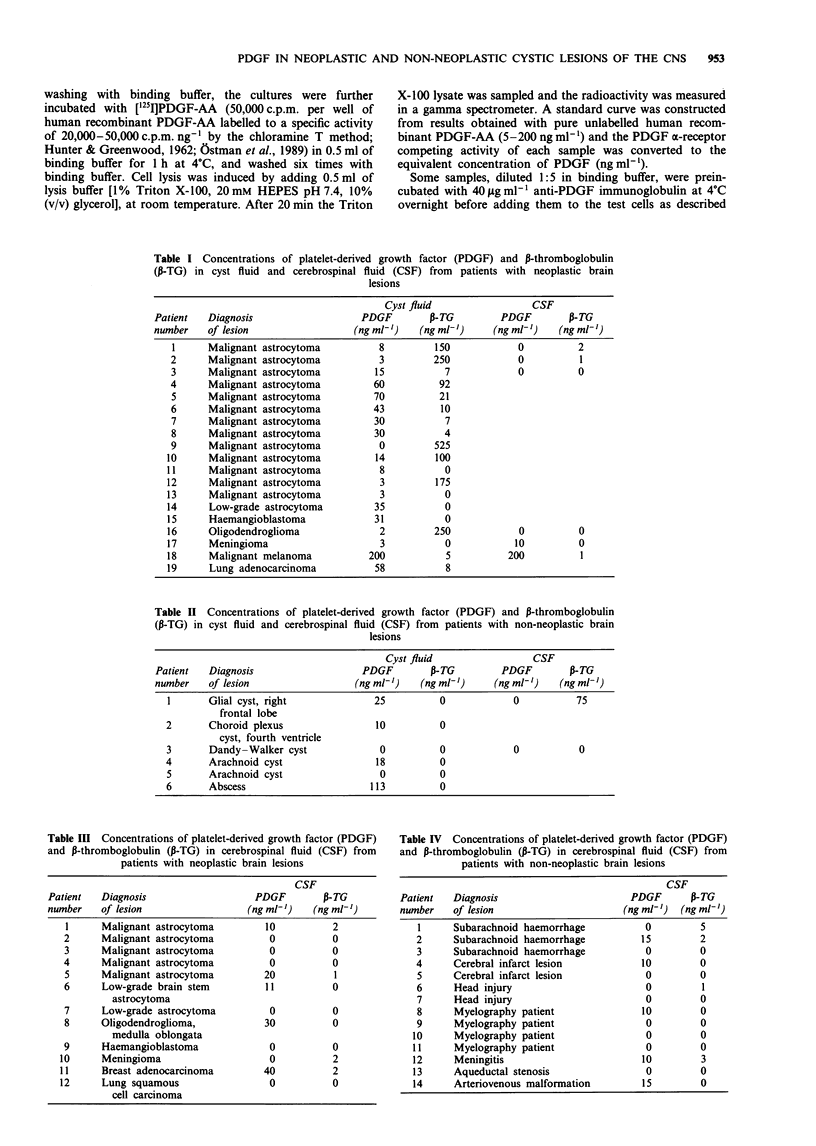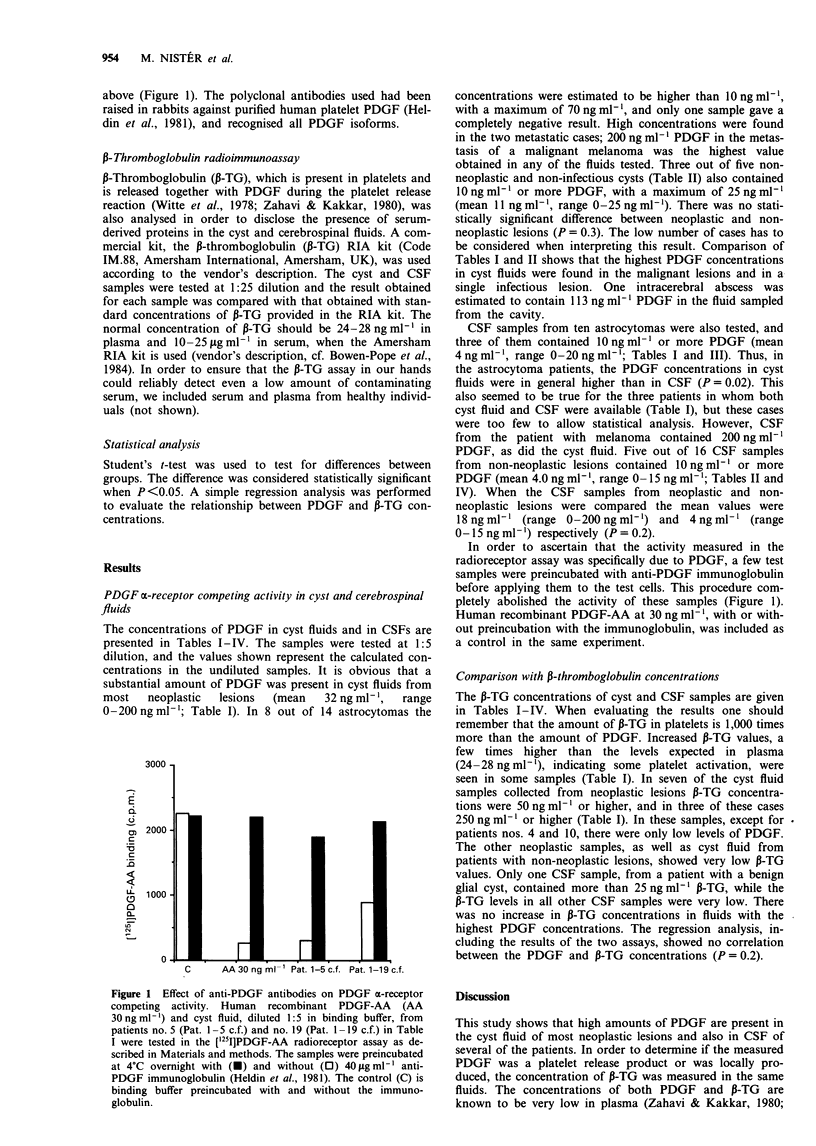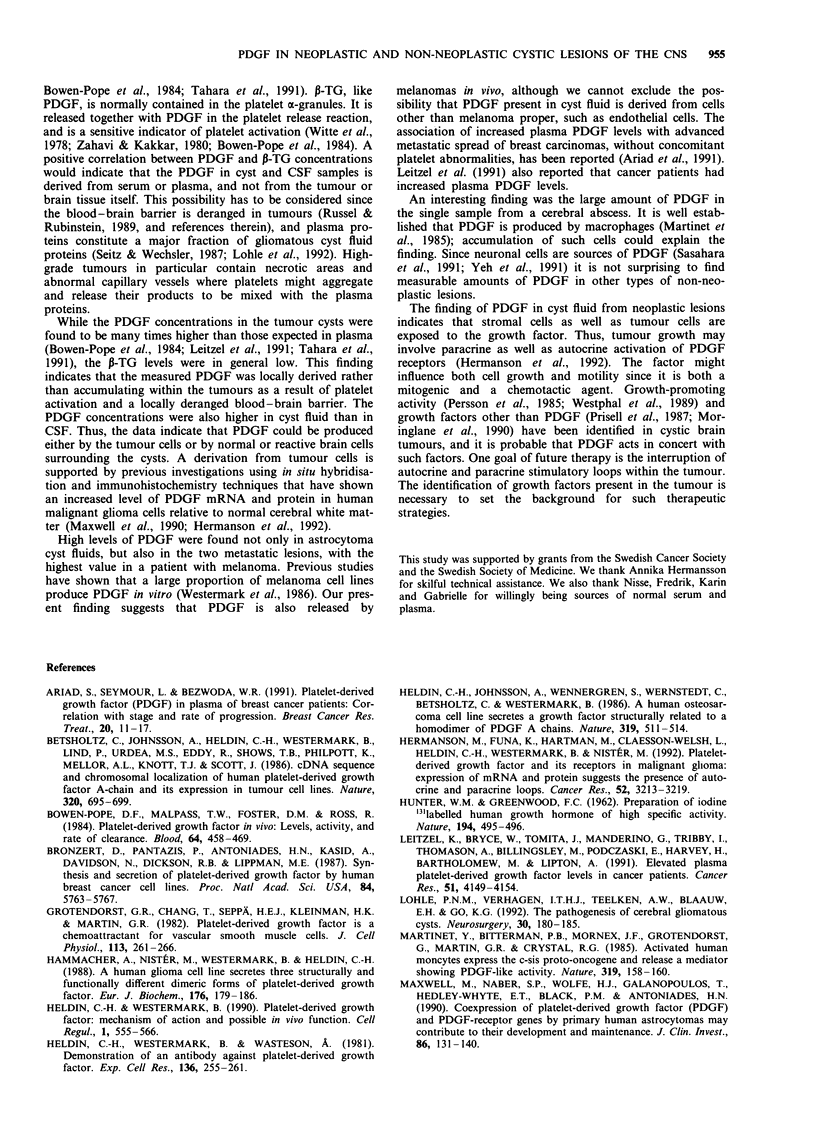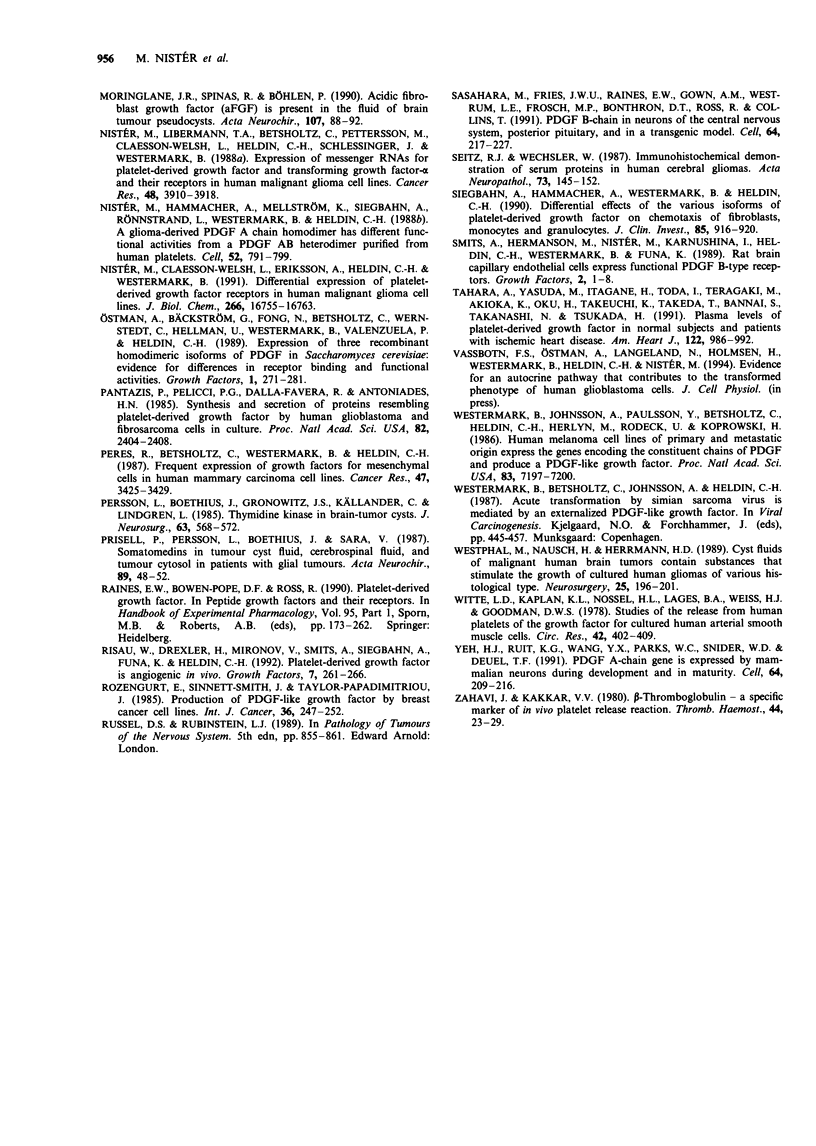# Platelet-derived growth factor (PDGF) in neoplastic and non-neoplastic cystic lesions of the central nervous system and in the cerebrospinal fluid.

**DOI:** 10.1038/bjc.1994.184

**Published:** 1994-05

**Authors:** M. Nistér, P. Enblad, G. Bäckström, T. Söderman, L. Persson, C. H. Heldin, B. Westermark

**Affiliations:** Department of Pathology, University of Uppsala, University Hospital, Sweden.

## Abstract

The aim of this study was to determine the concentration of PDGF in vivo in neoplastic and non-neoplastic brain lesions. Fluid from cystic lesions and cerebrospinal fluid was tested in a radioreceptor assay that detects all described PDGF isoforms. High concentration of PDGF were found in cyst fluids from several astrocytomas, one metastatic melanoma, one metastatic lung adenocarcinoma and one intracerebral abscess. The PDGF concentrations were several times higher than the levels known to be required for maximal PDGF effects on cells in vitro. PDGF could also be detected in some non-neoplastic lesions, especially one intracerebral abscess. The finding of high amounts of PDGF in neoplastic lesions strongly supports the possibility that PDGF can be a mediator of tumour and stromal cell growth and motility in vivo. Comparison of PDGF and beta-thromboglobulin concentrations in the same fluids strongly indicates that the PDGF protein is locally produced rather than a result of platelet activation and derangement of the blood-brain barrier.


Br. J. Cancer (1994), 69, 952-956 © Macmillan Press Ltd., 1994

### Platelet-derived growth factor (PDGF) in neoplastic and non-neoplastic cystic lesions of the central nervous system and in the cerebrospinal fluid

M. Nistér[1], P. Enblad[2], G. Bäckström[3], T. Söderman[1], L. Persson[2], C.-H. Heldin[3] & B. Westermark[1]

*Departments of [1]Pathology and [2]Neurosurgery, University of Uppsala, University Hospital, S-751 85 Uppsala, Sweden; [3]Ludwig Institute for Cancer Research, Biomedical Center, S-751 23 Uppsala, Sweden.*

**Summary** The aim of this study was to determine the concentration of PDGF *in vivo* in neoplastic and non-neoplastic brain lesions. Fluid from cystic lesions and cerebrospinal fluid was tested in a radioreceptor assay that detects all described PDGF isoforms. High concentrations of PDGF were found in cyst fluids from several astrocytomas, one metastatic melanoma, one metastatic lung adenocarcinoma and one intracerebral abscess. The PDGF concentrations were several times higher than the levels known to be required for maximal PDGF effects on cells *in vitro*. PDGF could also be detected in some non-neoplastic lesions, especially one intracerebral abscess. The finding of high amounts of PDGF in neoplastic lesions strongly supports the possibility that PDGF can be a mediator of tumour and stromal cell growth and motility *in vivo*. Comparison of PDGF and β-thromboglobulin concentrations in the same fluids strongly indicates that the PDGF protein is locally produced rather than a result of platelet activation and derangement of the blood-brain barrier.

Platelet-derived growth factor (PDGF) was originally recognised as a serum growth factor for fibroblasts, vascular smooth muscle cells and glial cells in culture (reviewed in Heldin & Westermark, 1990; Raines *et al.*, 1990). PDGF also influences the growth of brain capillary vessels (Smits *et al.*, 1989), and is a chemotactic and angiogenic agent (Grotendorst *et al.*, 1982; Siegbahn *et al.*, 1990; Risau *et al.*, 1992). PDGF in serum originates from platelet α-granules, and more recently it was realised that neuronal cells of the central nervous system (CNS) constitute another important source of PDGF *in vivo* (Sasahara *et al.*, 1991; Yeh *et al.*, 1991). Structurally, PDGF is a 30 kDa dimer of two homologous disulphide-bonded polypeptide chains denoted A and B, which are encoded by different genes. All three possible isoforms of PDGF have been identified and purified, namely PDGF-AA, PDGF-BB and PDGF-AB (reviewed in Heldin & Westermark, 1990; Raines *et al.*, 1990). These bind to two different but structurally related membrane receptors; all three dimeric forms of PDGF bind to the α-receptor, whereas the β-receptor has high affinity only for PDGF-BB and lower affinity for PDGF-AB. Thus, binding of [$^{125}$I]PDGF-AA to the α-receptor is competitively inhibited by all described PDGF isoforms.

Several experimental findings suggsest that PDGF might play a role in the pathogenesis of human tumours (for review see Westermark *et al.*, 1987). Several human tumour cell lines, e.g. glioma (Nistér *et al.*, 1991), sarcoma (Pantazis *et al.*, 1985; Betsholtz *et al.*, 1986), melanoma (Westermark *et al.*, 1986) and carcinoma (Rozengurt *et al.*, 1985; Bronzert *et al.*, 1987; Peres *et al.*, 1987) cell lines, produce PDGF in culture. Malignant glioma cell lines express both PDGF A- and B-chain genes or only PDGF-A (Nistér *et al.*, 1988*a*, 1991); however, they mainly secrete PDGF-AA into the extracellular medium (Hammacher *et al.*, 1988; Nistér *et al.*, 1988*b*), as do melanoma (Westermark *et al.*, 1986) and sarcoma (Betsholtz *et al.*, 1986; Heldin *et al.*, 1986) cell lines. Human glioma cell lines also express PDGF receptors, so that autocrine PDGF stimulation of these cells is possible (Nistér *et al.*, 1991). The growth of at least some glioma cell lines *in vitro* can actually be dependent on an autocrine PDGF loop (Vassbotn *et al.*, 1994).

It is thus possible that PDGF is one of the factors that drives the proliferation and migration of spontaneously occurring human primary and metastatic tumour cells within the CNS, as well as the vascular proliferation necessary for the growth of these lesions. The aim of this study was to determine whether PDGF is present in neoplastic cystic brain lesions of the CNS. The concentrations of PDGF in the tumour cyst fluids and in fluid from non-neoplastic control lesions were measured in a radioreceptor assay that detects all described isoforms of PDGF.

Correspondence: M. Nistér, Department of Pathology, University Hospital, S-751 85 Uppsala, Sweden.
Received 14 May 1993; and in revised form 25 November 1993.

### Materials and methods

### *Specimens*

Cyst fluids were obtained at surgery from 19 neoplastic (13 malignant astrocytomas, one low-grade astrocytoma, one oligodendroglioma, one haemangioblastoma, one meningioma, one metastatic malignant melanoma and one metastatic pulmonary adenocarcinoma; Table I) and six non-neoplastic cystic brain lesions (two arachnoid cysts, one glial cyst in the right frontal lobe, one Dandy-Walker cyst, one choroid plexus cyst in the fourth ventricle and one abscess; Table II). Cerebrospinal fluid (CSF) was collected from some of these patients, either by lumbar puncture or by ventricular puncture (Tables I and II). CSF was also obtained from 26 additional patients, of whom 12 had neoplastic lesions (five malignant astrocytomas and two low-grade astrocytomas, one meningioma, one haemangioblastoma, one oligodendroglioma, one metastatic mammary adencarcinoma and one metastatic squamous cell lung carcinoma; Table III) and 14 had non-neoplastic lesions (three patients with subarachnoid haemorrhage, two with cerebral infarction, two with head injury, one with meningitis, one with arteriovenous malformation, and one with hydrocephalus due to aqueductal stenosis). In addition, lumbar CSF was obtained from four patients undergoing myelography for suspected lumbar disc disease (Table IV). The CSF and cyst fluids were immediately centrifuged at 900 *g* and the supernatants frozen at −20°C until required for analysis.

### *Assay for PDGF α-receptor competing activity*

The concentrations of PDGF were measured indirectly by using an assay for PDGF α-receptor competing activity. Human foreskin fibroblasts, AG 1523, were seeded in 12 well plates, grown to confluence and washed once with binding buffer (phosphate-buffered saline containing 1 mg of bovine serum albumin, 0.01 mg ml$^{-1}$ calcium chloride dihydrate and 0.01 mg ml$^{-1}$ magnesium sulphate heptahydrate). The cells were incubated at 4°C with the test fluids (diluted 1:5 in binding buffer to a total volume of 0.5 ml) for 1.5 h. After washing with binding buffer, the cultures were further incubated with [$^{125}$I]PDGF-AA (50,000 c.p.m. per well of human recombinant PDGF-AA labelled to a specific activity of 20,000–50,000 c.p.m. $ng^{-1}$ by the chloramine T method; Hunter & Greenwood, 1962; Östman *et al.*, 1989) in 0.5 ml of binding buffer for 1 h at 4°C, and washed six times with binding buffer. Cell lysis was induced by adding 0.5 ml of lysis buffer [1% Triton X-100, 20 mM HEPES pH 7.4, 10% (v/v) glycerol], at room temperature. After 20 min the Triton X-100 lysate was sampled and the radioactivity was measured in a gamma spectrometer. A standard curve was constructed from results obtained with pure unlabelled human recombinant PDGF-AA (5–200 ng $ml^{-1}$) and the PDGF α-receptor competing activity of each sample was converted to the equivalent concentration of PDGF (ng $ml^{-1}$).

Some samples, diluted 1:5 in binding buffer, were preincubated with 40 μg $ml^{-1}$ anti-PDGF immunoglobulin at 4°C overnight before adding them to the test cells as described

**Table I** Concentrations of platelet-derived growth factor (PDGF) and β-thromboglobulin (β-TG) in cyst fluid and cerebrospinal fluid (CSF) from patients with neoplastic brain lesions

| | | *Cyst fluid* | | *CSF* | |
|---|---|---|---|---|---|
| *Patient number* | *Diagnosis of lesion* | *PDGF (ng ml⁻¹)* | *β-TG (ng ml⁻¹)* | *PDGF (ng ml⁻¹)* | *β-TG (ng ml⁻¹)* |
| 1 | Malignant astrocytoma | 8 | 150 | 0 | 2 |
| 2 | Malignant astrocytoma | 3 | 250 | 0 | 1 |
| 3 | Malignant astrocytoma | 15 | 7 | 0 | 0 |
| 4 | Malignant astrocytoma | 60 | 92 | | |
| 5 | Malignant astrocytoma | 70 | 21 | | |
| 6 | Malignant astrocytoma | 43 | 10 | | |
| 7 | Malignant astrocytoma | 30 | 7 | | |
| 8 | Malignant astrocytoma | 30 | 4 | | |
| 9 | Malignant astrocytoma | 0 | 525 | | |
| 10 | Malignant astrocytoma | 14 | 100 | | |
| 11 | Malignant astrocytoma | 8 | 0 | | |
| 12 | Malignant astrocytoma | 3 | 175 | | |
| 13 | Malignant astrocytoma | 3 | 0 | | |
| 14 | Low-grade astrocytoma | 35 | 0 | | |
| 15 | Haemangioblastoma | 31 | 0 | | |
| 16 | Oligodendroglioma | 2 | 250 | 0 | 0 |
| 17 | Meningioma | 3 | 0 | 10 | 0 |
| 18 | Malignant melanoma | 200 | 5 | 200 | 1 |
| 19 | Lung adenocarcinoma | 58 | 8 | | |

**Table II** Concentrations of platelet-derived growth factor (PDGF) and β-thromboglobulin (β-TG) in cyst fluid and cerebrospinal fluid (CSF) from patients with non-neoplastic brain lesions

| | | *Cyst fluid* | | *CSF* | |
|---|---|---|---|---|---|
| *Patient number* | *Diagnosis of lesion* | *PDGF (ng ml⁻¹)* | *β-TG (ng ml⁻¹)* | *PDGF (ng ml⁻¹)* | *β-TG (ng ml⁻¹)* |
| 1 | Glial cyst, right frontal lobe | 25 | 0 | 0 | 75 |
| 2 | Choroid plexus cyst, fourth ventricle | 10 | 0 | | |
| 3 | Dandy–Walker cyst | 0 | 0 | 0 | 0 |
| 4 | Arachnoid cyst | 18 | 0 | | |
| 5 | Arachnoid cyst | 0 | 0 | | |
| 6 | Abscess | 113 | 0 | | |

**Table III** Concentrations of platelet-derived growth factor (PDGF) and β-thromboglobulin (β-TG) in cerebrospinal fluid (CSF) from patients with neoplastic brain lesions

| | | *CSF* | |
|---|---|---|---|
| *Patient number* | *Diagnosis of lesion* | *PDGF (ng ml⁻¹)* | *β-TG (ng ml⁻¹)* |
| 1 | Malignant astrocytoma | 10 | 2 |
| 2 | Malignant astrocytoma | 0 | 0 |
| 3 | Malignant astrocytoma | 0 | 0 |
| 4 | Malignant astrocytoma | 0 | 0 |
| 5 | Malignant astrocytoma | 20 | 1 |
| 6 | Low-grade brain stem astrocytoma | 11 | 0 |
| 7 | Low-grade astrocytoma | 0 | 0 |
| 8 | Oligodendroglioma, medulla oblongata | 30 | 0 |
| 9 | Haemangioblastoma | 0 | 0 |
| 10 | Meningioma | 0 | 2 |
| 11 | Breast adenocarcinoma | 40 | 2 |
| 12 | Lung squamous cell carcinoma | 0 | 0 |

**Table IV** Concentrations of platelet-derived growth factor (PDGF) and β-thromboglobulin (β-TG) in cerebrospinal fluid (CSF) from patients with non-neoplastic brain lesions

| | | *CSF* | |
|---|---|---|---|
| *Patient number* | *Diagnosis of lesion* | *PDGF (ng ml⁻¹)* | *β-TG (ng ml⁻¹)* |
| 1 | Subarachnoid haemorrhage | 0 | 5 |
| 2 | Subarachnoid haemorrhage | 15 | 2 |
| 3 | Subarachnoid haemorrhage | 0 | 0 |
| 4 | Cerebral infarct lesion | 10 | 0 |
| 5 | Cerebral infarct lesion | 0 | 0 |
| 6 | Head injury | 0 | 1 |
| 7 | Head injury | 0 | 0 |
| 8 | Myelography patient | 10 | 0 |
| 9 | Myelography patient | 0 | 0 |
| 10 | Myelography patient | 0 | 0 |
| 11 | Myelography patient | 0 | 0 |
| 12 | Meningitis | 10 | 3 |
| 13 | Aqueductal stenosis | 0 | 0 |
| 14 | Arteriovenous malformation | 15 | 0 |

above (Figure 1). The polyclonal antibodies used had been raised in rabbits against purified human platelet PDGF (Heldin *et al.*, 1981), and recognised all PDGF isoforms.

*β-Thromboglobulin radioimmunoassay*

β-Thromboglobulin (β-TG), which is present in platelets and is released together with PDGF during the platelet release reaction (Witte *et al.*, 1978; Zahavi & Kakkar, 1980), was also analysed in order to disclose the presence of serum-derived proteins in the cyst and cerebrospinal fluids. A commercial kit, the β-thromboglobulin (β-TG) RIA kit (Code IM.88, Amersham International, Amersham, UK), was used according to the vendor's description. The cyst and CSF samples were tested at 1:25 dilution and the result obtained for each sample was compared with that obtained with standard concentrations of β-TG provided in the RIA kit. The normal concentration of β-TG should be 24–28 ng ml$^{-1}$ in plasma and 10–25 μg ml$^{-1}$ in serum, when the Amersham RIA kit is used (vendor's description, cf. Bowen-Pope *et al.*, 1984). In order to ensure that the β-TG assay in our hands could reliably detect even a low amount of contaminating serum, we included serum and plasma from healthy individuals (not shown).

*Statistical analysis*

Student's *t*-test was used to test for differences between groups. The difference was considered statistically significant when $P<0.05$. A simple regression analysis was performed to evaluate the relationship between PDGF and β-TG concentrations.

### Results

*PDGF α-receptor competing activity in cyst and cerebrospinal fluids*

The concentrations of PDGF in cyst fluids and in CSFs are presented in Tables I–IV. The samples were tested at 1:5 dilution, and the values shown represent the calculated concentrations in the undiluted samples. It is obvious that a substantial amount of PDGF was present in cyst fluids from most neoplastic lesions (mean 32 ng ml$^{-1}$, range 0–200 ng ml$^{-1}$; Table I). In 8 out of 14 astrocytomas the

**Figure 1** Effect of anti-PDGF antibodies on PDGF α-receptor competing activity. Human recombinant PDGF-AA (AA 30 ng ml$^{-1}$) and cyst fluid, diluted 1:5 in binding buffer, from patients no. 5 (Pat. 1–5 c.f.) and no. 19 (Pat. 1–19 c.f.) in Table I were tested in the [$^{125}$I]PDGF-AA radioreceptor assay as described in Materials and methods. The samples were preincubated at 4°C overnight with (■) and without (□) 40 μg ml$^{-1}$ anti-PDGF immunoglobulin (Heldin *et al.*, 1981). The control (C) is binding buffer preincubated with and without the immunoglobulin.

concentrations were estimated to be higher than 10 ng ml$^{-1}$, with a maximum of 70 ng ml$^{-1}$, and only one sample gave a completely negative result. High concentrations were found in the two metastatic cases; 200 ng ml$^{-1}$ PDGF in the metastasis of a malignant melanoma was the highest value obtained in any of the fluids tested. Three out of five non-neoplastic and non-infectious cysts (Table II) also contained 10 ng ml$^{-1}$ or more PDGF, with a maximum of 25 ng ml$^{-1}$ (mean 11 ng ml$^{-1}$, range 0–25 ng ml$^{-1}$). There was no statistically significant difference between neoplastic and non-neoplastic lesions ($P = 0.3$). The low number of cases has to be considered when interpreting this result. Comparison of Tables I and II shows that the highest PDGF concentrations in cyst fluids were found in the malignant lesions and in a single infectious lesion. One intracerebral abscess was estimated to contain 113 ng ml$^{-1}$ PDGF in the fluid sampled from the cavity.

CSF samples from ten astrocytomas were also tested, and three of them contained 10 ng ml$^{-1}$ or more PDGF (mean 4 ng ml$^{-1}$, range 0–20 ng ml$^{-1}$; Tables I and III). Thus, in the astrocytoma patients, the PDGF concentrations in cyst fluids were in general higher than in CSF ($P = 0.02$). This also seemed to be true for the three patients in whom both cyst fluid and CSF were available (Table I), but these cases were too few to allow statistical analysis. However, CSF from the patient with melanoma contained 200 ng ml$^{-1}$ PDGF, as did the cyst fluid. Five out of 16 CSF samples from non-neoplastic lesions contained 10 ng ml$^{-1}$ or more PDGF (mean 4.0 ng ml$^{-1}$, range 0–15 ng ml$^{-1}$; Tables II and IV). When the CSF samples from neoplastic and non-neoplastic lesions were compared the mean values were 18 ng ml$^{-1}$ (range 0–200 ng ml$^{-1}$) and 4 ng ml$^{-1}$ (range 0–15 ng ml$^{-1}$) respectively ($P = 0.2$).

In order to ascertain that the activity measured in the radioreceptor assay was specifically due to PDGF, a few test samples were preincubated with anti-PDGF immunoglobulin before applying them to the test cells. This procedure completely abolished the activity of these samples (Figure 1). Human recombinant PDGF-AA at 30 ng ml$^{-1}$, with or without preincubation with the immunoglobulin, was included as a control in the same experiment.

*Comparison with β-thromboglobulin concentrations*

The β-TG concentrations of cyst and CSF samples are given in Tables I–IV. When evaluating the results one should remember that the amount of β-TG in platelets is 1,000 times more than the amount of PDGF. Increased β-TG values, a few times higher than the levels expected in plasma (24–28 ng ml$^{-1}$), indicating some platelet activation, were seen in some samples (Table I). In seven of the cyst fluid samples collected from neoplastic lesions β-TG concentrations were 50 ng ml$^{-1}$ or higher, and in three of these cases 250 ng ml$^{-1}$ or higher (Table I). In these samples, except for patients nos. 4 and 10, there were only low levels of PDGF. The other neoplastic samples, as well as cyst fluid from patients with non-neoplastic lesions, showed very low β-TG values. Only one CSF sample, from a patient with a benign glial cyst, contained more than 25 ng ml$^{-1}$ β-TG, while the β-TG levels in all other CSF samples were very low. There was no increase in β-TG concentrations in fluids with the highest PDGF concentrations. The regression analysis, including the results of the two assays, showed no correlation between the PDGF and β-TG concentrations ($P = 0.2$).

### Discussion

This study shows that high amounts of PDGF are present in the cyst fluid of most neoplastic lesions and also in CSF of several of the patients. In order to determine if the measured PDGF was a platelet release product or was locally produced, the concentration of β-TG was measured in the same fluids. The concentrations of both PDGF and β-TG are known to be very low in plasma (Zahavi & Kakkar, 1980;

Bowen-Pope *et al.*, 1984; Tahara *et al.*, 1991). β-TG, like PDGF, is normally contained in the platelet α-granules. It is released together with PDGF in the platelet release reaction, and is a sensitive indicator of platelet activation (Witte *et al.*, 1978; Zahavi & Kakkar, 1980; Bowen-Pope *et al.*, 1984). A positive correlation between PDGF and β-TG concentrations would indicate that the PDGF in cyst and CSF samples is derived from serum or plasma, and not from the tumour or brain tissue itself. This possibility has to be considered since the blood-brain barrier is deranged in tumours (Russel & Rubinstein, 1989, and references therein), and plasma proteins constitute a major fraction of gliomatous cyst fluid proteins (Seitz & Wechsler, 1987; Lohle *et al.*, 1992). High-grade tumours in particular contain necrotic areas and abnormal capillary vessels where platelets might aggregate and release their products to be mixed with the plasma proteins.

While the PDGF concentrations in the tumour cysts were found to be many times higher than those expected in plasma (Bowen-Pope *et al.*, 1984; Leitzel *et al.*, 1991; Tahara *et al.*, 1991), the β-TG levels were in general low. This finding indicates that the measured PDGF was locally derived rather than accumulating within the tumours as a result of platelet activation and a locally deranged blood-brain barrier. The PDGF concentrations were also higher in cyst fluid than in CSF. Thus, the data indicate that PDGF could be produced either by the tumour cells or by normal or reactive brain cells surrounding the cysts. A derivation from tumour cells is supported by previous investigations using *in situ* hybridisation and immunohistochemistry techniques that have shown an increased level of PDGF mRNA and protein in human malignant glioma cells relative to normal cerebral white matter (Maxwell *et al.*, 1990; Hermanson *et al.*, 1992).

High levels of PDGF were found not only in astrocytoma cyst fluids, but also in the two metastatic lesions, with the highest value in a patient with melanoma. Previous studies have shown that a large proportion of melanoma cell lines produce PDGF *in vitro* (Westermark *et al.*, 1986). Our present finding suggests that PDGF is also released by melanomas *in vivo*, although we cannot exclude the possibility that PDGF present in cyst fluid is derived from cells other than melanoma proper, such as endothelial cells. The association of increased plasma PDGF levels with advanced metastatic spread of breast carcinomas, without concomitant platelet abnormalities, has been reported (Ariad *et al.*, 1991). Leitzel *et al.* (1991) also reported that cancer patients had increased plasma PDGF levels.

An interesting finding was the large amount of PDGF in the single sample from a cerebral abscess. It is well established that PDGF is produced by macrophages (Martinet *et al.*, 1985); accumulation of such cells could explain the finding. Since neuronal cells are sources of PDGF (Sasahara *et al.*, 1991; Yeh *et al.*, 1991) it is not surprising to find measurable amounts of PDGF in other types of non-neoplastic lesions.

The finding of PDGF in cyst fluid from neoplastic lesions indicates that stromal cells as well as tumour cells are exposed to the growth factor. Thus, tumour growth may involve paracrine as well as autocrine activation of PDGF receptors (Hermanson *et al.*, 1992). The factor might influence both cell growth and motility since it is both a mitogenic and a chemotactic agent. Growth-promoting activity (Persson *et al.*, 1985; Westphal *et al.*, 1989) and growth factors other than PDGF (Prisell *et al.*, 1987; Moringlane *et al.*, 1990) have been identified in cystic brain tumours, and it is probable that PDGF acts in concert with such factors. One goal of future therapy is the interruption of autocrine and paracrine stimulatory loops within the tumour. The identification of growth factors present in the tumour is necessary to set the background for such therapeutic strategies.

This study was supported by grants from the Swedish Cancer Society and the Swedish Society of Medicine. We thank Annika Hermansson for skilful technical assistance. We also thank Nisse, Fredrik, Karin and Gabrielle for willingly being sources of normal serum and plasma.

### References

ARIAD, S., SEYMOUR, L. & BEZWODA, W.R. (1991). Platelet-derived growth factor (PDGF) in plasma of breast cancer patients: Correlation with stage and rate of progression. *Breast Cancer Res. Treat.*, **20**, 11-17.

BETSHOLTZ, C., JOHNSSON, A., HELDIN, C.-H., WESTERMARK, B., LIND, P., URDEA, M.S., EDDY, R., SHOWS, T.B., PHILPOTT, K., MELLOR, A.L., KNOTT, T.J. & SCOTT, J. (1986). cDNA sequence and chromosomal localization of human platelet-derived growth factor A-chain and its expression in tumour cell lines. *Nature*, **320**, 695-699.

BOWEN-POPE, D.F., MALPASS, T.W., FOSTER, D.M. & ROSS, R. (1984). Platelet-derived growth factor *in vivo*: Levels, activity, and rate of clearance. *Blood*, **64**, 458-469.

BRONZERT, D., PANTAZIS, P., ANTONIADES, H.N., KASID, A., DAVIDSON, N., DICKSON, R.B. & LIPPMAN, M.E. (1987). Synthesis and secretion of platelet-derived growth factor by human breast cancer cell lines. *Proc. Natl Acad. Sci. USA*, **84**, 5763-5767.

GROTENDORST, G.R., CHANG, T., SEPPÄ, H.E.J., KLEINMAN, H.K. & MARTIN, G.R. (1982). Platelet-derived growth factor is a chemoattractant for vascular smooth muscle cells. *J. Cell Physiol.*, **113**, 261-266.

HAMMACHER, A., NISTÉR, M., WESTERMARK, B. & HELDIN, C.-H. (1988). A human glioma cell line secretes three structurally and functionally different dimeric forms of platelet-derived growth factor. *Eur. J. Biochem.*, **176**, 179-186.

HELDIN, C.-H. & WESTERMARK, B. (1990). Platelet-derived growth factor: mechanism of action and possible *in vivo* function. *Cell Regul.*, **1**, 555-566.

HELDIN, C.-H., WESTERMARK, B. & WASTESON, Å. (1981). Demonstration of an antibody against platelet-derived growth factor. *Exp. Cell Res.*, **136**, 255-261.

HELDIN, C.-H., JOHNSSON, A., WENNERGREN, S., WERNSTEDT, C., BETSHOLTZ, C. & WESTERMARK, B. (1986). A human osteosarcoma cell line secretes a growth factor structurally related to a homodimer of PDGF A chains. *Nature*, **319**, 511-514.

HERMANSON, M., FUNA, K., HARTMAN, M., CLAESSON-WELSH, L., HELDIN, C.-H., WESTERMARK, B. & NISTÉR, M. (1992). Platelet-derived growth factor and its receptors in malignant glioma: expression of mRNA and protein suggests the presence of autocrine and paracrine loops. *Cancer Res.*, **52**, 3213-3219.

HUNTER, W.M. & GREENWOOD, F.C. (1962). Preparation of iodine $^{131}$labelled human growth hormone of high specific activity. *Nature*, **194**, 495-496.

LEITZEL, K., BRYCE, W., TOMITA, J., MANDERINO, G., TRIBBY, I., THOMASON, A., BILLINGSLEY, M., PODCZASKI, E., HARVEY, H., BARTHOLOMEW, M. & LIPTON, A. (1991). Elevated plasma platelet-derived growth factor levels in cancer patients. *Cancer Res.*, **51**, 4149-4154.

LOHLE, P.N.M., VERHAGEN, I.T.H.J., TEELKEN, A.W., BLAAUW, E.H. & GO, K.G. (1992). The pathogenesis of cerebral gliomatous cysts. *Neurosurgery*, **30**, 180-185.

MARTINET, Y., BITTERMAN, P.B., MORNEX, J.F., GROTENDORST, G., MARTIN, G.R. & CRYSTAL, R.G. (1985). Activated human moncytes express the c-sis proto-oncogene and release a mediator showing PDGF-like activity. *Nature*, **319**, 158-160.

MAXWELL, M., NABER, S.P., WOLFE, H.J., GALANOPOULOS, T., HEDLEY-WHYTE, E.T., BLACK, P.M. & ANTONIADES, H.N. (1990). Coexpression of platelet-derived growth factor (PDGF) and PDGF-receptor genes by primary human astrocytomas may contribute to their development and maintenance. *J. Clin. Invest.*, **86**, 131-140.

MORINGLANE, J.R., SPINAS, R. & BÖHLEN, P. (1990). Acidic fibroblast growth factor (aFGF) is present in the fluid of brain tumour pseudocysts. *Acta Neurochir.*, **107**, 88–92.

NISTÉR, M., LIBERMANN, T.A., BETSHOLTZ, C., PETTERSSON, M., CLAESSON-WELSH, L., HELDIN, C.-H., SCHLESSINGER, J. & WESTERMARK, B. (1988*a*). Expression of messenger RNAs for platelet-derived growth factor and transforming growth factor-α and their receptors in human malignant glioma cell lines. *Cancer Res.*, **48**, 3910–3918.

NISTÉR, M., HAMMACHER, A., MELLSTRÖM, K., SIEGBAHN, A., RÖNNSTRAND, L., WESTERMARK, B. & HELDIN, C.-H. (1988*b*). A glioma-derived PDGF A chain homodimer has different functional activities from a PDGF AB heterodimer purified from human platelets. *Cell*, **52**, 791–799.

NISTÉR, M., CLAESSON-WELSH, L., ERIKSSON, A., HELDIN, C.-H. & WESTERMARK, B. (1991). Differential expression of platelet-derived growth factor receptors in human malignant glioma cell lines. *J. Biol. Chem.*, **266**, 16755–16763.

ÖSTMAN, A., BÄCKSTRÖM, G., FONG, N., BETSHOLTZ, C., WERNSTEDT, C., HELLMAN, U., WESTERMARK, B., VALENZUELA, P. & HELDIN, C.-H. (1989). Expression of three recombinant homodimeric isoforms of PDGF in *Saccharomyces cerevisiae*: evidence for differences in receptor binding and functional activities. *Growth Factors*, **1**, 271–281.

PANTAZIS, P., PELICCI, P.G., DALLA-FAVERA, R. & ANTONIADES, H.N. (1985). Synthesis and secretion of proteins resembling platelet-derived growth factor by human glioblastoma and fibrosarcoma cells in culture. *Proc. Natl Acad. Sci. USA*, **82**, 2404–2408.

PERES, R., BETSHOLTZ, C., WESTERMARK, B. & HELDIN, C.-H. (1987). Frequent expression of growth factors for mesenchymal cells in human mammary carcinoma cell lines. *Cancer Res.*, **47**, 3425–3429.

PERSSON, L., BOETHIUS, J., GRONOWITZ, J.S., KÄLLANDER, C. & LINDGREN, L. (1985). Thymidine kinase in brain-tumor cysts. *J. Neurosurg.*, **63**, 568–572.

PRISELL, P., PERSSON, L., BOETHIUS, J. & SARA, V. (1987). Somatomedins in tumour cyst fluid, cerebrospinal fluid, and tumour cytosol in patients with glial tumours. *Acta Neurochir.*, **89**, 48–52.

RAINES, E.W., BOWEN-POPE, D.F. & ROSS, R. (1990). Platelet-derived growth factor. In Peptide growth factors and their receptors. In *Handbook of Experimental Pharmacology*, Vol. 95, Part 1, Sporn, M.B. & Roberts, A.B. (eds), pp. 173–262. Springer: Heidelberg.

RISAU, W., DREXLER, H., MIRONOV, V., SMITS, A., SIEGBAHN, A., FUNA, K. & HELDIN, C.-H. (1992). Platelet-derived growth factor is angiogenic *in vivo*. *Growth Factors*, **7**, 261–266.

ROZENGURT, E., SINNETT-SMITH, J. & TAYLOR-PAPADIMITRIOU, J. (1985). Production of PDGF-like growth factor by breast cancer cell lines. *Int. J. Cancer*, **36**, 247–252.

RUSSEL, D.S. & RUBINSTEIN, L.J. (1989). In *Pathology of Tumours of the Nervous System*. 5th edn, pp. 855–861. Edward Arnold: London.

SASAHARA, M., FRIES, J.W.U., RAINES, E.W., GOWN, A.M., WESTRUM, L.E., FROSCH, M.P., BONTHRON, D.T., ROSS, R. & COLLINS, T. (1991). PDGF B-chain in neurons of the central nervous system, posterior pituitary, and in a transgenic model. *Cell*, **64**, 217–227.

SEITZ, R.J. & WECHSLER, W. (1987). Immunohistochemical demonstration of serum proteins in human cerebral gliomas. *Acta Neuropathol.*, **73**, 145–152.

SIEGBAHN, A., HAMMACHER, A., WESTERMARK, B. & HELDIN, C.-H. (1990). Differential effects of the various isoforms of platelet-derived growth factor on chemotaxis of fibroblasts, monocytes and granulocytes. *J. Clin. Invest.*, **85**, 916–920.

SMITS, A., HERMANSON, M., NISTÉR, M., KARNUSHINA, I., HELDIN, C.-H., WESTERMARK, B. & FUNA, K. (1989). Rat brain capillary endothelial cells express functional PDGF B-type receptors. *Growth Factors*, **2**, 1–8.

TAHARA, A., YASUDA, M., ITAGANE, H., TODA, I., TERAGAKI, M., AKIOKA, K., OKU, H., TAKEUCHI, K., TAKEDA, T., BANNAI, S., TAKANASHI, N. & TSUKADA, H. (1991). Plasma levels of platelet-derived growth factor in normal subjects and patients with ischemic heart disease. *Am. Heart J.*, **122**, 986–992.

VASSBOTN, F.S., ÖSTMAN, A., LANGELAND, N., HOLMSEN, H., WESTERMARK, B., HELDIN, C.-H. & NISTÉR, M. (1994). Evidence for an autocrine pathway that contributes to the transformed phenotype of human glioblastoma cells. *J. Cell Physiol.* (in press).

WESTERMARK, B., JOHNSSON, A., PAULSSON, Y., BETSHOLTZ, C., HELDIN, C.-H., HERLYN, M., RODECK, U. & KOPROWSKI, H. (1986). Human melanoma cell lines of primary and metastatic origin express the genes encoding the constituent chains of PDGF and produce a PDGF-like growth factor. *Proc. Natl Acad. Sci. USA*, **83**, 7197–7200.

WESTERMARK, B., BETSHOLTZ, C., JOHNSSON, A. & HELDIN, C.-H. (1987). Acute transformation by simian sarcoma virus is mediated by an externalized PDGF-like growth factor. In *Viral Carcinogenesis*. Kjelgaard, N.O. & Forchhammer, J. (eds), pp. 445-457. Munksgaard: Copenhagen.

WESTPHAL, M., NAUSCH, H. & HERRMANN, H.D. (1989). Cyst fluids of malignant human brain tumors contain substances that stimulate the growth of cultured human gliomas of various histological type. *Neurosurgery*, **25**, 196–201.

WITTE, L.D., KAPLAN, K.L., NOSSEL, H.L., LAGES, B.A., WEISS, H.J. & GOODMAN, D.W.S. (1978). Studies of the release from human platelets of the growth factor for cultured human arterial smooth muscle cells. *Circ. Res.*, **42**, 402–409.

YEH, H.J., RUIT, K.G., WANG, Y.X., PARKS, W.C., SNIDER, W.D. & DEUEL, T.F. (1991). PDGF A-chain gene is expressed by mammalian neurons during development and in maturity. *Cell*, **64**, 209–216.

ZAHAVI, J. & KAKKAR, V.V. (1980). β-Thromboglobulin – a specific marker of *in vivo* platelet release reaction. *Thromb. Haemost.*, **44**, 23–29.